# Evolution of complex organelles in ocelloid-bearing dinoflagellates

**DOI:** 10.1093/pnasnexus/pgag287

**Published:** 2026-08-27

**Authors:** Elizabeth C Cooney, Corey C Holt, Gordon Lax, Patrick J Keeling

**Affiliations:** Department of Botany, University of British Columbia, Vancouver, BC, Canada V6T 1Z4; Department of Botany, University of British Columbia, Vancouver, BC, Canada V6T 1Z4; Department of Botany, University of British Columbia, Vancouver, BC, Canada V6T 1Z4; Department of Botany, University of British Columbia, Vancouver, BC, Canada V6T 1Z4

**Keywords:** warnowiids, plastid, dinoflagellate, single-cell transcriptomics, photosystems

## Abstract

Warnowiid dinoflagellates are known for possessing two of the most complex structures known in single cells: multiprojectile nematocysts and the camera eye-like ocelloid. The rarity of warnowiids and their intractability in culture have stymied efforts to understand the function and role of these structures. Recent single-cell transcriptome sequencing efforts revealed that the plastid within the ocelloid complex (analogous to the retina in an animal eye) may have lost photosystem II (PSII), while retaining photosystem I as a putative light-sensing mechanism. However, this finding was produced from only 18 cells across five genera and did not include many of the known lineages within this group. To interrogate this finding, we generated single-cell transcriptomes for 12 additional species, including from two previously unsampled genera, collected from subarctic and tropical environments. A search for photosynthesis-related genes across all taxa supports previous findings that PSII has likely been lost in the warnowiid ocelloid plastid. Additionally, our updated phylogenomic analysis reveals that warnowiids can be divided into two major subclades based on the possession of nematocysts: armed and unarmed. Moreover, ocelloid size, shape, position, and gene expression are distinct between armed and unarmed warnowiids and between genera in some cases, indicating that ocelloid function may differ between lineages and this may be linked to differences in energy and nutritional strategy.

Significance statement Complex structures in warnowiid dinoflagellates—i.e. the camera eye-like ocelloid and complex nematocysts—have garnered interest for over a century, but due to the rarity and intractability of these cells in culture, this group has proven difficult to study. Single-cell transcriptomics has provided a new avenue for exploration of this group, recently revealing insights about the unique biology of the ocelloid plastid. This study significantly expands the sampling of diverse warnowiids, introducing several new lineages to the phylogenomic analysis of warnowiids. This exploration reveals new evolutionary insights into ocelloid form and nematocyst presence, suggesting that ocelloid function may vary across the warnowiids.

## Introduction

Warnowiid dinoflagellates possess among the most complex structures observed within a single cell, with subcellular components reminiscent of whole organs in multicellular animals. In particular, the multiprojectile nematocysts used for hunting prey and the ocelloid, a structure with a striking resemblance to an animal camera eye, confounded early observers, some of whom were convinced these cells must have phagocytosed part of a decaying cnidarian (1). The fact that these features evolved independently from animals in the single-celled warnowiids has garnered significant interest. However, because members of this group are rare and have never been successfully cultivated, robust biochemical and gene expression experiments have remained stubbornly unfeasible.

Despite these limitations, advances have been made toward determining the evolutionary history and function of specialized warnowiid structures. Detailed explorations of ocelloid ultrastructure performed on uncultivated species using transmission electron microscopy and FIB-SEM revealed that the cornea and retina analogs (the latter referred to as the “retinal body” in warnowiids) are derived from highly modified mitochondria and plastids, respectively (2, 3). Early phylogenetics using ribosomal genes could not resolve relationships in the group but revealed that warnowiids were members of Gymnodiniales and closely related to nematocyst-carrying *Polykrikos* (4, 5). More recently, transcriptomic data generated from single cells confirmed that these two nematocyst-carrying lineages both branch in the Gymnodiniales but are, in fact, not sisters. More interestingly, transcriptome data also revealed the absence of photosystem II (PSII) in ocelloid plastids, leading to the model that the remaining photosynthetic machinery had been repurposed for light sensing in the ocelloid (6).

These insights are a major advance for a completely uncultivated lineage but also come from only 18 cells across five genera, representing only a fraction of the diversity observed in warnowiids. Moreover, transcriptomes are inherently incomplete, representing only genes that are expressed at the time of sampling and, especially for genes with low expression, there is always the possibility of undersampling, particularly in single-cell approaches. This kind of data is therefore robust to claim a gene or pathway is present, but to say one is absent requires additional scrutiny. In this way, the absence of PSII transcripts is key to understanding the function of the ocelloid, and additional support for this is important. While efforts to cultivate warnowiids continue to be unsuccessful, expanding single-cell transcriptomic sampling to encompass greater warnowiid diversity is a more feasible approach that will not only further test the hypothesis that PSII has truly been lost in the ocelloid, but also has the potential to expand the known diversity of the group as well.

Here, we expand warnowiid transcriptomic data more than 3-fold with cells collected from both subarctic and tropical environments. We include two previously unsampled genera and show that the warnowiids are split into two distinct morphological clades—one with nematocysts and one without. Our investigation further supports that PSII is likely absent in warnowiid ocelloid plastids and importantly reveals contrasts in plastidial ATP synthase gene expression across genera that might provide more insights into the conversion of the organelle from photosynthesis to sensory functions.

## Results and discussion

### Identification of cells

A total of 44 diverse individual warnowiid cells were collected from tropical and subarctic environments: 21 off the island of Curaçao and 23 from the Gulf of Alaska (Table S1, Fig. S1, videos: https://doi.org/10.5683/SP4/DANYQG). Aside from *Erythropsidinium scarlatinum*, which was easily recognized, many cells were not identifiable from morphology alone, as physical features varied widely. Taxonomic affiliations for these were first determined based on phylogenetic affinity of 18S and 28S rRNA gene sequences extracted from assembled transcriptomes and subsequently from a multigene phylogenomic analysis.

Ribosomal gene phylogenies (Figs. S2 and S3) do not yield a well-supported topology for this group (warnowiids do not even branch together in 18S rRNA trees) but are nonetheless informative for comparing new cells with already identified species. The new cells fell within numerous warnowiid subgroups. *Erythropsidinium scarlatinum* sequences branch sister to *Erythropsidinium* from the Salish Sea in Canada (6, 7), hereafter referred to as *Erythropsidinium nutkana* sp. nov. (see Supplementary information for taxonomic descriptions). Some of the *Cyklopsia* sequences we generated cluster closely with *Cyklopsia gemma*, indicating they are members of this species, consistent with their general shape, hyaline appearance, and small cell size. Other *Cyklopsia* cells were larger and had pink pigmentation, and these form a distinct clade sister to *C. gemma* in both 18S and 28S rRNA gene phylogenies, as well as the multigene phylogeny. These cells match the morphological description previously assigned to *Nematopsides vigilans* (8, 9), but due to their close relationship to *C. gemma*, we propose to transfer this species to the genus *Cyklopsia*.

**Figure 1:**
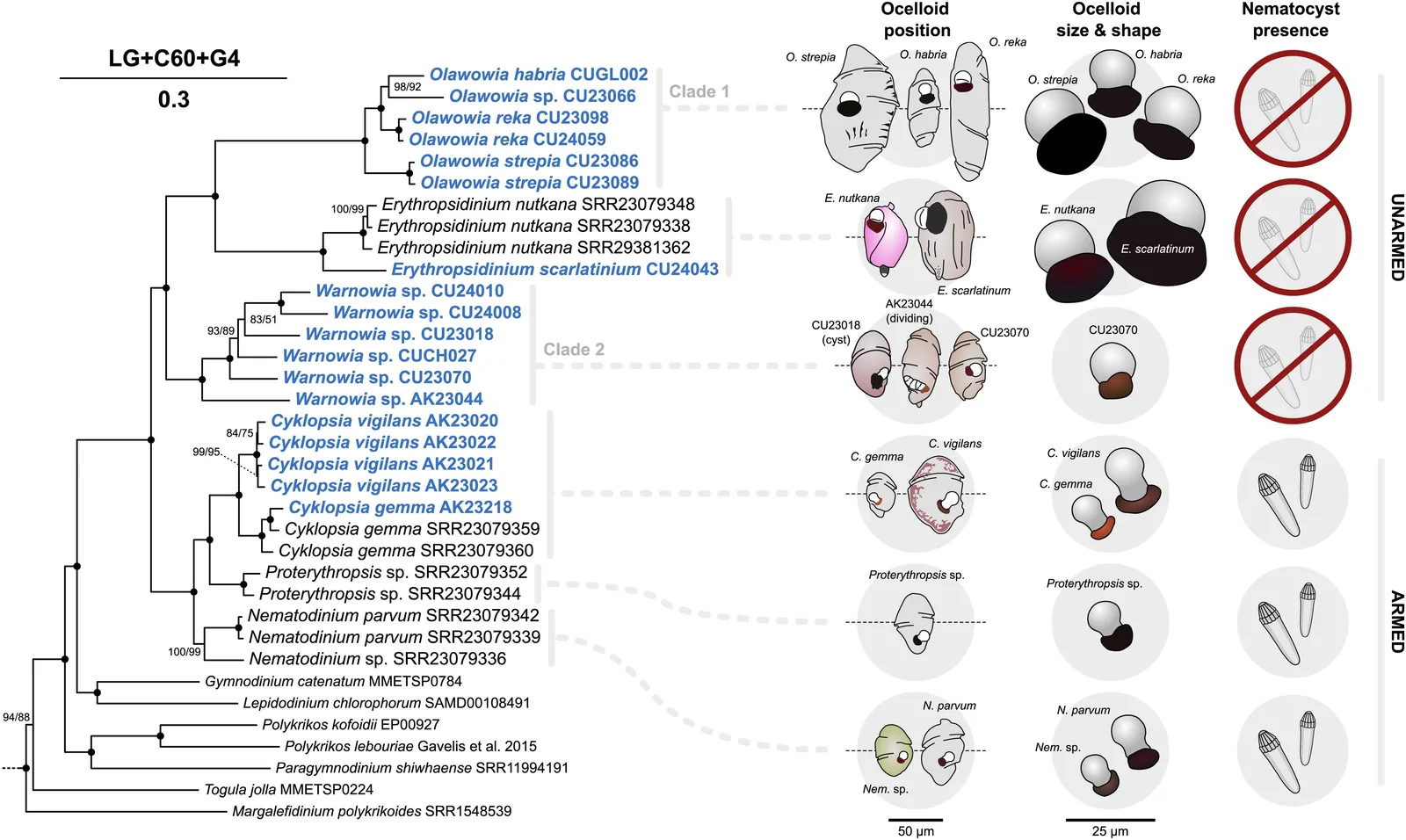
ML multigene phylogeny and character mapping of warnowiids. The Gymnodiniales *sensu stricto* clade shown is a subset of a phylogenomic tree generated from 225 conserved genes (for the full tree: https://doi.org/10.5683/SP4/DANYQG). Node values represent bootstrap support (1,000 ultrafast/100 PMSF nonparametric) with dots indicating a value of 100 in both analyses. Scale bar at the top left shows the estimated number of amino acid substitutions per site with the initial model used to generate this topology. Transcriptomes collected in this study are bold, colored blue. For each warnowiid genus, schematic depictions of ocelloid position, ocelloid size and shape, and the presence of nematocysts are shown. Some cell cartoons were borrowed from figures in Cooney et al. (6).

The remaining sequences did not branch closely with *Erythropsidinium* or *Cyklopsia*, and generally clustered with sequences annotated in GenBank as “*Warnowia* sp.,” but organisms with this name are nonmonophyletic. One group of sequences form a distinct long-branching clade in both phylogenies, the placement of which is not well-supported (Figs. S2 and S3). A phylogenomic analysis of 225 orthologous genes from 85 operational taxonomic units (OTUs) was performed to resolve these groups and their relationships within the warnowiid clade (Fig. 1). In the multigene tree, the placement of *Erythropsidinium* and *Cyklopsia* cells are consistent with those observed in the rRNA gene phylogenies. Moreover, in this more robust phylogeny, the cells that formed long-branching clades in rRNA phylogenies are fully supported as sisters to *Erythropsidinium* in the multigene tree, and all other cells also form a strongly supported clade that branches sister to this group and *Erythropsidinium*.

Cells in these two groups share some characteristics with one another. Specifically, both have elongated cells with an offset cingulum that encircles the cell more than two times. However, ocelloids in the first clade are generally located in the anterior half of the cell or at the midline, and are anteriorly facing, while ocelloids in the second clade lie in the posterior half of the cell, facing laterally or anterolaterally. Early descriptions of the genus *Warnowia* (syn. *Pouchetia*) included cells that likely belonged to both of these groups, judging by the placement and orientation of ocelloids (1, 9, 10). The type species of *Warnowia*, *Warnowia fusus*, is depicted in its initial description as a cell with two opposite-facing lenses on the ocelloid, making it impossible to determine ocelloid placement, since the cell was likely undergoing division (10, 11). We now show *Warnowia* is nonmonophyletic and, therefore, designate the first subgroup (with long-branching rRNA) as a new genus, *Olawowia* gen. nov., with three new species, *Olawowia habria* sp. nov., *Olawowia reka* sp. nov., and *Olawowia strepia* sp. nov. Another cell previously identified as *Warnowia* sp. (6) was also recently reclassified as *Nematodinium parvum* (12).

### Character mapping and distinguishing features

Like many athecate dinoflagellates, identifying species-defining characteristics in the warnowiids has historically been a challenge. Because of their rarity and apparent intractability in culture, life cycles of cells cannot easily be observed to eliminate transitional features of division or encystment from stable trophic states as possible distinguishing features. Indeed, observations suggest that many species described in early literature are conspecific, and morphology within species is more plastic than previously assumed (5, 13, 14). By generating a robust phylogenomic analysis that resolves the relationships between diverse warnowiid genera, we are now able to better compare and contrast group characteristics (Fig. 1).

Ocelloid location at, or anterior to, the midline of the cell seems to be a consistent feature of the *Olawowia* + *Erythropsidinium* clade, as opposed to all other warnowiids where the ocelloid lies in the posterior half of the cell (Fig. 1). However, ocelloids are likely the first component to transform during cytokinesis and may move position before changes in the cell body are detectable under light microscopy, making this feature unreliable on its own for identification (13, 14). Similarly, the orientation of the ocelloid in *Olawowia* + *Erythropsidinium* is sometimes anterior-facing rather than laterally or anterolaterally facing, as seems to be consistent in other warnowiids. However, this orientation might also be adjustable, as *Erythropsidinium* cells have been observed rotating their ocelloids (13), possibly explaining why *E. scarlatinum* cells presented in this study had laterally directed ocelloids at the time of collection. Cells likely belonging to *Olawowia* have also been observed with anterolaterally tilted ocelloids, suggesting ocelloid orientation is also not static in this genus (13).

Ocelloid shape, unlike location and orientation, follows a stricter pattern. Specifically, the lenses of *Olawowia*, *Erythropsidinium*, and *Warnowia* are almost spherical, like an orb set into the retinal body pocket, giving the whole complex a width barely smaller than its length. In contrast, lenses in *Cyklopsia*, *Proterythropsis*, and *Nematodinium* are elongated and pyriform, tapering gradually from the bulbous tip, or with an abrupt shoulder forming a slightly narrowed neck extending to the retinal body. The retinal body is nearly the same size or larger than the lens in *Olawowia* and *Erythropsidinium* ocelloids, whereas the lens is considerably larger than the retinal body in all other genera. Unfortunately, only one *Warnowia* cell with a fully formed ocelloid was collected in this study (CU23070), and all other cells from this genus were in various states of division or encystment. It is therefore important that more sampling be conducted within this group to determine if the ocelloid features described here are consistent across *Warnowia* species.

Nematocyst presence matched the phylogenomic pattern of ocelloid lens shape. While most collected individuals within *Cyklopsia*, *Proterythropsis*, and *Nematodinium* were observed to carry nematocysts, *Olawowia*, *Erythropsidinium*, and *Warnowia* lacked these structures (Fig. S1). Nematocysts are not always visibly present in cells that might possess them, so nematocyst absence is possibly not a reliable diagnostic for identifying warnowiids. However, the pattern of presence and absence we observed fits exactly with the strongly supported phylogenomic tree, suggesting that the ability to make these complex structures functionally divides warnowiids into two major phylogenomic lineages: armed and unarmed.

### Photosynthetic gene expression and divergence

**Figure 2:**
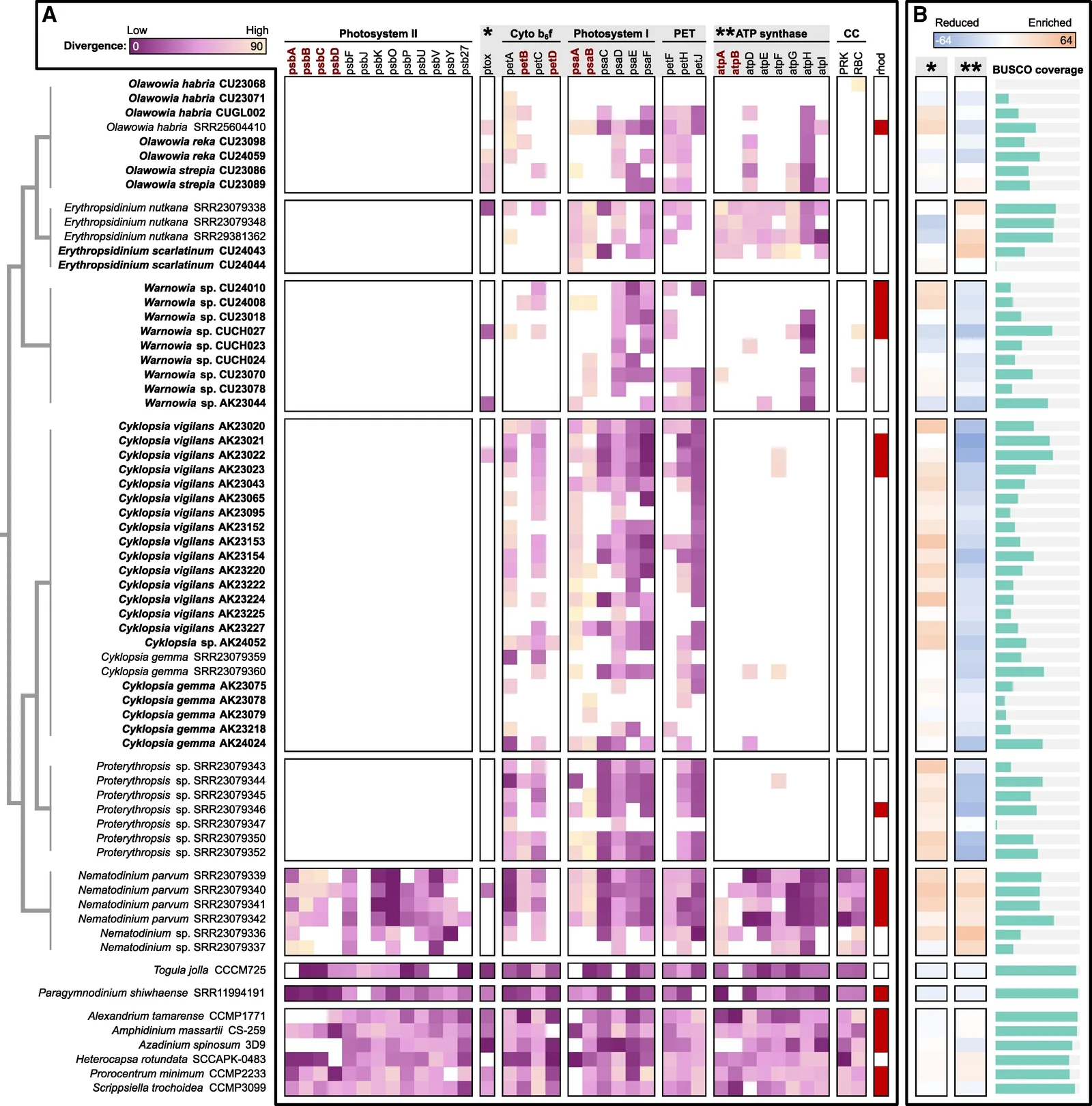
Presence, divergence, and relative frequency of photosynthesis-related gene transcripts in warnowiids. Phylogenomic relationships between warnowiid genera from Fig. 1 are depicted in a cladogram. Individual cells collected in this study are bolded. A) Colored boxes indicate transcript presence for genes of photosystem II, cytochrome b_6_f, photosystem I, photosynthetic electron transport (PET), ATP synthase, the Calvin cycle (CC), and rhodopsin. Hue variations represent divergence via the average difference in BLAST TS of the corresponding species’ recovered transcript sequences from a homolog of the close relative, *G. catenatum*. In this color value spectrum, purple represents low divergence, and yellow represents high divergence. Bolded read column headers are genes typically encoded in dinoflagellate peridinin plastid genomes. B) Relative representation in transcriptome of ptox + cytochrome b_6_f + photosystem I + PET (*), and ATP synthase (**) compared to BUSCO orthologs (alveolata_odb10). Bar graph shows the presence of BUSCO orthologs in each transcriptome.

**Figure 3:**
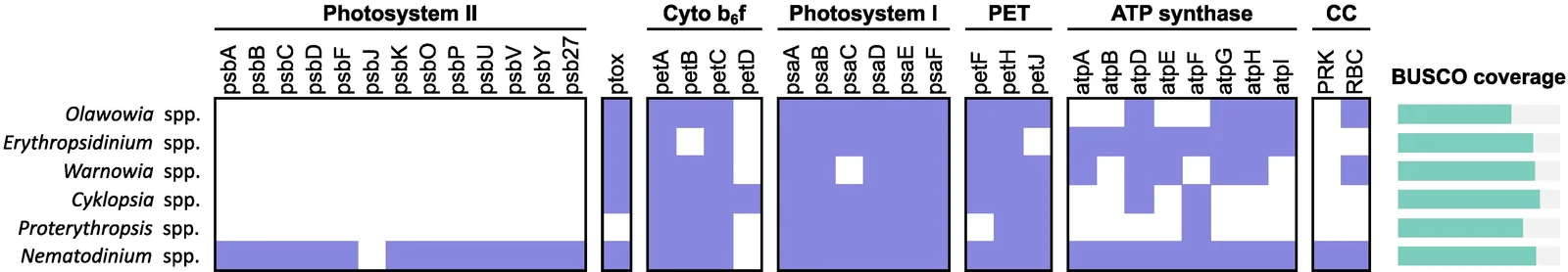
Genus-level summary of photosynthesis-related gene presence. Colored boxes indicate that at least one transcript was recovered from a single-cell transcriptome within the genus. Bar graph shows BUSCO coverage of all combined transcriptomic data available for each genus.

A search for genes associated with the photosynthetic pathways confirmed the complete absence of any gene associated with PSII in any of the taxa, except *Nematodinium*, which is known to still possess photosynthetic plastids in addition to the ocelloid plastid (Figs. 2 and 3). One cell (CU23078) contained some PSII transcripts, but these were determined to be contaminants from recently consumed prey (Fig. S4). The absence of PSII transcripts across so many cells strengthens the argument that this photosystem has been completely lost, and indeed recent work with *Symbiodinium* demonstrating spontaneous loss of PSII genes suggests this is a common starting point for photosynthesis reduction in dinoflagellates more broadly (15). In contrast, the cytochrome b_6_f, photosynthetic electron transport (PET), and photosystem I (PSI) complexes were all consistently represented in all warnowiid lineages, as seen previously (6), but we observed interesting inconsistencies in the expression of ATP synthase.

Genes encoding subunits of plastidial ATP (pATP) synthase are present in all genera, but the range of genes found appears dramatically different across groups (Fig. 2A). Within the unarmed warnowiids, *Erythropsidinium* expressed all components of this complex, but transcripts of several subunits were consistently absent from *Olawowia* and *Warnowia*, suggesting the complex in this lineage is reduced or functionally altered. Even more starkly, expression of the pATP synthase subunits was barely detectable in nonphotosynthetic armed warnowiid transcriptomes, with only *atpD* or *atpF* recovered from five out of the total 30 individual cells. Searches for five out of the eight pATP synthase genes recovered mitochondrial or vacuolar homologs in almost all warnowiid genera (Fig. S5). This raises the possibility that if pATP synthase has been mostly lost, ATP complexes from other cell compartments could function in the plastid. However, there is currently no evidence for these proteins being targeted to the plastid, and if pATP synthase is degraded in some species, it could also point to differences in ocelloid function across genera, and the high coverage of the collective transcriptome data for each combined genus suggests that in most cases, missing genes cannot be explained simply by undersampling (Fig. 3).

To interrogate whether paucity of pATP synthase transcripts in some genera was just the result of undersampling, the proportion of transcript presence vs. absence across pATP synthase genes was compared to presence vs. absence in BUSCO coverage estimates (Fig. 2B). This comparison was performed for pATP synthase (represented in our analysis by eight genes) as well as for the energy gradient-producing portion of the photosynthetic pathway, omitting PSII to avoid skewing results (i.e. plastoquinol-oxidizing plastid terminal oxidase [*ptox*], Cytochrome b_6_f, PSI, and PET—14 genes total). Generally, the latter was more enriched (compared to BUSCO coverage) across warnowiids, with a mix of enriched and reduced states in the unarmed warnowiids. In contrast, pATP synthase was enriched in *Erythropsidinium*, with equal or reduced representation in all other nonphotosynthetic warnowiids. The enrichment of pATP synthase in *Nematodinium* may be due to the presence of photosynthetic plastids in these cells in addition to the ocelloid plastid.

A search for twin-arginine translocation (TAT) genes was performed to determine whether the presence of this pathway in the transcriptomes suggested mechanistic coupling with pATP synthase. The TAT pathway transports folded proteins across the thylakoid membrane and is powered by proton motive force; therefore, pATP synthase activity may affect its function (16). Transcripts of *tatA*, *tatB*, and *tatC* were all recovered from the genera *Nematodinium*, *Cyklopsia*, and *Warnowia*, and only *tatB* was not recovered in *Erythropsidinium* and *Proterythropsis* (Fig. S5). TAT pathway genes were absent from collective *Olawowia* transcriptomic data, but given that this was the genus with the lowest overall coverage and these genes have low expression across the other genera, this absence is very possibly the result of undersampling. No notable differences in transcript presence could be detected between genera that would indicate that the TAT pathway was differentially linked to pATP synthase.

The scarcity of pATP synthase transcripts in armed warnowiids and the sparseness of typically highly expressed ribulose-1,5-bisphosphate carboxylase (RuBisCo, or RBC in Fig. 2) in all nonphotosynthetic warnowiids suggests these mechanisms might have unique functionality in these groups. Indeed, most of the gene sequences recovered for these components are divergent at the sequence level (Fig. 2A) and form long branches in single gene phylogenies (Fig. S6) compared to those of the close photosynthetic relatives, *Nematodinium* spp. and *Gymnodinium catenatum*. In contrast, two “typical” photosynthetic peridinin plastid-carrying members of Gymnodiniales *sensu stricto*, *Paragymnodinium shiwhaense*, and *Togula jolla* show less divergence of these sequences, despite having a more distant relationship to *G. catenatum* than the warnowiids (Fig. S6). RuBisCo was previously hypothesized to be completely absent in the nonphotosynthetic warnowiids (6), but we observed this gene is present in at least *Olawowia* and *Warnowia*. However, the extremely low expression of this gene and the continued lack of evidence for PSII and phosphoribulokinase (PRK) still suggest that conventional photosynthesis does not occur in these cells.

A comparison between all warnowiids and nonwarnowiid dinoflagellates analyzed here (including an assortment of non-Gymnodiniales *sensu stricto* species) reveals that certain photosynthesis-derived genes are particularly divergent in warnowiids. The transmembrane components of PSI that bind chlorophyll, *psaA* and *psaB*, are consistently divergent across all warnowiids, including *Nematodinium* (Fig. 2A). These proteins form a dimer that acts as the transmembrane core of the PSI complex, and the loss of either component would lead to loss of the complex (17, 18). Both genes are clearly retained and expressed in the ocelloid plastid, and their deviation may be linked to the unique thylakoid architecture in the ocelloid (3), as photosystem complexes are known to affect thylakoid membrane conformation (19). Compositional divergence could also indicate changes in the electron transfer or antenna anchoring roles of these proteins from their ancestral state.

Although rhodopsin was detected, transcripts are sporadically present across the warnowiids, going undetected in most cells (Fig. 2A). In some cells, rhodopsin transcripts were abundant, while only one or two transcripts were recovered in others (Fig. S7). Warnowiid rhodopsins fell into five distinct groups: type I proteorhodopsin, type II proteorhodopsin, cryptophyte-type sensory rhodopsin, archaea-type sensory rhodopsin, and heliorhodopsin, indicating horizontal acquisition from many sources (Fig. S7). *Warnowia* sp. CUCH027 expressed two different kinds of rhodopsin, but one of these was an archaea-type sensory rhodopsin, which we cannot rule out was contamination. Transcripts from *Cyklopsia vigilans* AK23021 and *O. habria* SRR25604410 were similarly ambiguous. Rhodopsin transcripts are conspicuously absent in all *Erythropsidinium*, which have the largest retinal bodies and highest average transcriptome coverage of all genera sampled. The distribution of rhodopsins in warnowiids suggests that they are not highly conserved and likely serve different functions depending on the rhodopsin type and the lineage.

### Nutritional strategy implications

The distinct character states of armed and unarmed warnowiids suggest there are differences in their nutritional strategies. Although the role of the ocelloid cannot yet be confirmed, it is thought this structure is involved in prey capture, either through prey selection, by detecting the polarized light passing through the condensed nuclei of dinoflagellate prey (3), or by general detection when a prey cell is in nematocyst firing range (20). The co-occurrence of elongated, pyriform ocelloids with nematocysts suggests these features are functionally linked, and perhaps the ocelloid does act as a companion mechanism to the nematocysts for the purposes of prey capture in this group. However, this association cannot explain the presence of ocelloids, which in many cases are larger and differently shaped in the unarmed warnowiids.

The difference in ocelloid shape between armed and unarmed warnowiids may provide clues about differences in function. In light and dark adaptation experiments, ocelloids of *Erythropsidinium* were shown to change shape, with the retinal body expanding to have greater surface area, forming a tighter cup and enveloping the lens in a nest-like manner when dark-adapted (21). Since the angle of light capture increases when the retinal body is more cupped around the lens, this may be an adaptation to capture more photons when light is limited. Notably, the ocelloids of *Olawowia* and *Erythropsidinium* during interphase are much more nest like than those of armed warnowiids, despite the fact that they live in high-light environments (Fig. 1). This conflicts with the assumption that all ocelloids are light sensors, given that the armed warnowiids in this study were only found in light-depleted environments, while *Olawowia* and *E. scarlatinum* inhabit high-light tropical and subtropical waters.

**Figure 4:**
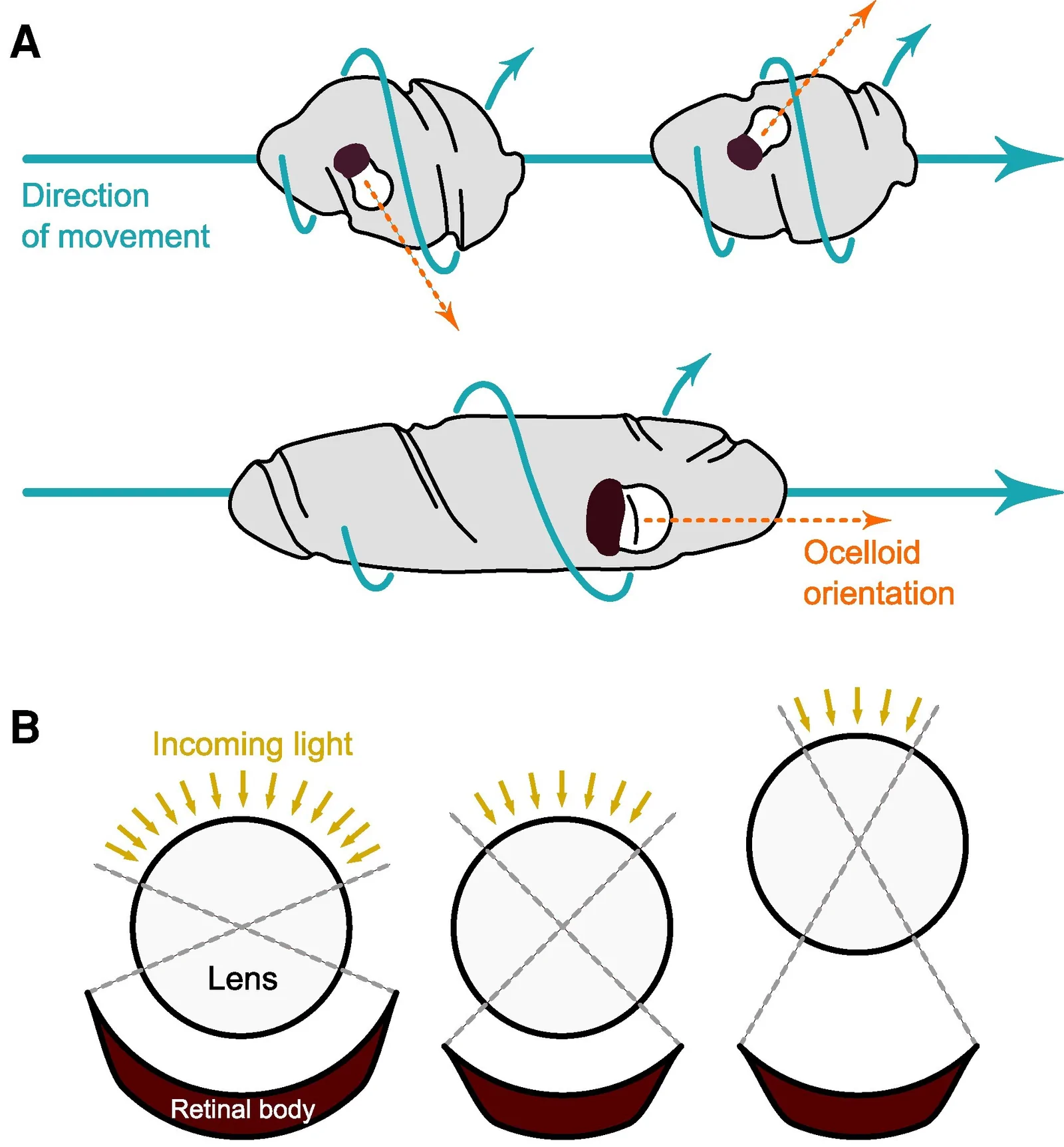
Schematics of ocelloid characteristic variation. A) Comparison of ocelloid directionality as a result of swimming in armed (top) and unarmed warnowiids that have anteriorly directed ocelloids (bottom). Solid arrows correspond to cell movement, dotted arrows indicate ocelloid direction. B) Range of directional light detection in ocelloids with lenses nested in a large retinal body (left), ocelloids with a narrower retinal body relative to lens size (middle), and ocelloids with a narrow retinal body and elongated lens (right). Arrows represent incoming light that can interact with the retinal body.

There is a strong case to be made that the role of ocelloids in armed warnowiids is directional light sensing for phototaxis and predation. In the Gulf of Alaska and the Salish Sea during spring, upwelling and wind mixing inject nutrients into the sunlit surface layer, causing phytoplankton blooms and providing ample food for predators like *Cyklopsia* and *Proterythropsis*. In this environment, light near the surface can attenuate quickly due to the accumulation of organic matter compared to the oligotrophic tropics, where light penetrates to greater depths (22). Since disparities in light during spring in the northern latitudes are directly linked to phytoplankton distribution, the ability to detect differences in light intensity and direction might enable a predatory cell to move toward more favorable prey conditions. The seemingly permanent tilt of the ocelloids in armed warnowiids may assist with directional detection, as this orientation enables a wide scan of the environment as the cell swims in a spiral motion (Fig. 4A). The elongated shape and smaller retinal bodies of armed warnowiid ocelloids are consistent with this, as these features narrow the range of light detection (Fig. 4B), enabling more precision in detecting light direction as the cell moves. A sudden disruption in light caused by a passing prey cell may also trigger the firing of nematocysts.

Compared to their armed counterparts, some features of unarmed warnowiids (particularly *Erythropsidinium* and *Olawowia*) and their ocelloids seem inconsistent with a light-sensing role for the purposes of predation. For example, although *Erythropsidinium* possesses a piston that is thought to facilitate prey capture, its ocelloid faces away from this structure and thus cannot detect an object within firing range the way ocelloids of armed warnowiids might (13, 20). The inflated size and complexity of *Erythropsidinium* and *Olawowia* ocelloids compared to armed warnowiids seems excessive for basic light sensing, especially considering the higher light exposure most of the former are subject to in mid-latitudes. Moreover, the deeper nesting of lenses in the retinal bodies of these unarmed genera should allow these ocelloids to capture photons from a broader directional range, reducing directional precision, possibly suggesting a role in light sensing for more general behavior, such as positioning the cell within the water column.

Perhaps the most compelling difference between warnowiid genera is in the relative expression of ATP synthase components. *Erythropsidinium* is the only nonphotosynthetic warnowiid genus in which plastidial ATP synthase is completely present and enriched in the transcriptome (Figs. 2A and 3), indicating that ATP production is still an important function of its ocelloid plastid. This raises the possibility that the *Erythropsidinium* ocelloid focuses light for energy production rather than to fulfill a sensory role. Given the unique characteristics of this genus, like the piston, it would be unsurprising to discover that these cells also possess unique nutritional or energy strategies, although retention of both photosystems would obviously have fulfilled this goal much more efficiently. *Erythropsidinium* is known to capture and consume prey using its piston (13), but this appendage is often absent, as observed in three out of the five cells included in this study (and others observed personally). Maintaining the piston is likely energy expensive, as evidenced by its encasing in a mitochondrial cuff (23), so the ability to absorb this appendage when food is scarce and continue producing ATP from sun energy would be advantageous.

In stark contrast, the scarcity of ATP synthase and the enrichment of PSI and accompanying machinery in nonphotosynthetic armed warnowiids (Figs. 2 and 3) suggest that the activity of the remaining photosystem is not primarily powering ATP synthesis and may be coupled with a different mechanism. This is supported by the divergence of the transcripts recovered, suggesting that there is less selective pressure to conserve these genes (Figs. 2A and S3). *Warnowia* and *Olawowia* express some ATP synthase genes more consistently than the armed genera, but the expression of this complex is reduced in these groups overall, and multiple components could not be detected in any transcriptome. These two genera may represent an intermediate stage of plastidial ATP synthase erosion mirroring ancestors of *Cyklopsia* and *Proterythropsis*.

Confoundingly, many of the features of a light-sensing ocelloid are also consistent with it being an energy capture mechanism. If an ocelloid solely senses light for phototaxis and prey capture, a wider, more cupped retinal body may increase the angle of detection, and the pigmented lipid droplets surrounding the retinal body are analogous to the stigma of other eyespots, which provide one-sided shading for directional light detection (24). Alternatively, if an ocelloid functions as a solar cell, a wider retinal body maximizes photon capture, and the lipid droplet screen may protect the rest of the cell from lens-focused light damage. The former hypothesis is a reasonable one, since light sensing is an ability present in many unicellular algae. However, this is typically achieved with much simpler eyespots and fails to explain the exceptional complexity that has arisen in the warnowiids. For this reason, it is important to consider other potential roles the ocelloid might fulfill until further experimentation can be performed.

## Materials and methods

### Sample collection

Samples were collected during four field excursions in 2023 and 2024 (Table S1). Subarctic sampling was conducted aboard the R/V Sikuliaq on two spring cruises for the Northern Gulf of Alaska Long Term Ecological Research project. Tropical samples were collected near the CarMaBi Research Station in Curaçao. Plankton samples were collected using a handheld 21-µm mesh plankton net and then inspected on a Leica DMIL inverted microscope. Cells were isolated using a microcapillary pipet, washed in 0.2 µm filtered seawater and imaged with a Sony aIII7 or Sony 7s camera. After imaging, cells were placed into 2 µL of lysis buffer and frozen for preservation.

### Sequencing and transcriptome assembly

Generation of cDNA was performed following the Smart-seq2 protocol, eluting bead-cleaned cDNA in ultrapure water (25). Libraries were prepared by the UBC Bioinformatics and Sequencing consortium using Illumina DNA Prep and sequenced on the NextSeq2000 platform. Just two samples were sequenced on the NovaSeqX platform at The Centre for Applied Genomics, Toronto (Ontario). Forward and reverse raw reads were trimmed using Cutadapt v3.2 (26) and assembled using rnaSPAdes v3.15.5 (27). Assemblies were translated into peptides by identifying open reading frames with Transdecoder and using BLASTP v2.15.0 (*e*-value ≤1*e*-5) (28) to search sequences against known proteins in the UniProt/Swiss-Prot database (29), helping to identify and retain all protein-coding sequences during annotation. Quality of all assemblies was assessed using the alveolate_odb10 database in BUSCO v5.4.3 (30, 31). Nucleotide and peptide assemblies can be found at https://doi.org/10.5683/SP4/DANYQG.

### Phylogenetic and phylogenomic analyses

Small subunit ribosomal RNA (18S rRNA) and large subunit ribosomal RNA (28S rRNA) gene sequences were extracted from nucleotide assemblies using Barrnap v0.9 (32). Sequences were aligned with the curated collections of warnowiid and other dinoflagellate sequences from Cooney et al. (6) using MAFFT v7.481 (33). Alignments were trimmed in TrimAl v1.4 with a gap threshold of 0.3 (34). A phylogenetic tree was then generated using FastTree v2.1.11 (35), and contaminant sequences were identified based on phylogenetic affinity and eliminated. The most complete sequence was selected from the remaining sequences for each individual cell sample and all others were eliminated. Remaining sequences were realigned and trimmed following the same steps described above. For both 18S and 28S, a maximum likelihood (ML) analysis was performed in IQ-TREE v2.1.0 (36) using the best-fit model GTR + F + I + G4 selected by ModelFinder (37) and 1,000 ultrafast bootstraps (38).

To perform a multigene phylogenomic analysis, 263 curated conserved orthologous gene collections were used as queries in BLASTP searches (*e*-value ≤1*e*-5) against all transcriptomes generated in this study. Hits were parsed to retain sequences with an *e*-value ≤1*e*-20 and query coverage >50%. To find and remove sequence extensions, these selected sequences were used as queries to perform a BLASTP search against the UniProtKB/Swiss-Prot database (29). The hits resulting from this search were used to identify the mature protein portion of our sequences, so that any excess could be removed. The resulting trimmed sequences were aligned with their original query files from the 263 gene set using MAFFT-LINS-I, alignments were trimmed with TrimAl (gap threshold = 0.8), and trees were generated using FastTree. Each tree was visually inspected to identify and remove paralogs, isoforms, and contaminants, leaving at most one representative sequence for each cell sample in each of the conserved 263 genes. Selected sequences were combined with their respective ortholog collections, which were aligned using MAFFT-LINS-I and trimmed with TrimAl (gap threshold = 0.8). Trimmed alignments were parsed and concatenated using SCaFoS v4.55 (39), relevant taxa were selected for inclusion in the final phylogeny, and then only orthologs present in ≥60% of these taxa were concatenated to produce the final alignment. For transcriptomes generated in this study, only the highest-coverage transcriptomes of each distinct lineage were included, to avoid the destabilizing effect that low-coverage transcriptomes can have on downstream analysis. An ML analysis was performed on this alignment in IQ-TREE using the empirical profile mixture model LG + C60 + G4 (40), omitting the amino acid frequency “F-class” (41), and 1,000 ultrafast bootstraps. A second analysis was then performed on the same alignment using the model LG + C60 + G4 PMSF (42), using the previous tree as a guide and 100 nonparametric bootstraps.

### Plastid gene search and divergence

Searches for transcripts associated with photosynthetic pathway components (i.e. for PSI and PSII, *ptox*, cytochrome b_6_f, PET, ATP synthase, and Calvin cycle), rhodopsin, and the TAT pathway were performed with BLASTP (*e*-value ≤1*e*-5), using curated collections of orthologous sequences from diverse OTUs as queries for each gene (6). All hits were retained and aligned with their respective queries using MAFFT. Alignments were trimmed with TrimAl (gap threshold = 0.8), and trees were generated from trimmed alignments using FastTree. Trees were visually inspected to identify and remove contaminant sequences. After cleaning, sequence collections were aligned with MAFFT-LINS-I and trimmed in TrimAl (gap threshold = 0.01). An ML analysis was performed on each alignment using IQ-TREE with the empirical profile mixture model LG + C20 + G4 and 1,000 ultrafast bootstraps.

To assess relative divergence of warnowiid sequences from typical photosynthetic peridinin plastid genes, sequences from the transcriptomes sequenced here and previously published warnowiid transcriptomes were compared to those of a close relative to the warnowiids, *G. catenatum*. Since cells collected in this study are not true replicates nor biologically independent (as many were collected on the same day from the same net tows), statistical tests could not be performed. Instead, for each relevant photosynthesis-related gene, alignment scores of all noncontaminant homologs from warnowiids were compared against a *G. catenatum* homolog using BLASTP in NCBI (default parameters, including BLOSUM62 scoring matrix). Two more “typical” photosynthetic relatives, *P. shiwhaense* and *T. jolla*, were also searched in this way to compare nonocelloid-bearing species to the warnowiids. For every pairwise comparison, the BLAST total score (TS) was recorded, and in cases where a transcriptome yielded more than one sequence (due to isoforms or paralogs), their TS were averaged, generating a single representative value for each specimen. These values were normalized using the following equation: $n=(|\mathrm{TS}-TS_{\max}|÷TS_{\max})\times 100$, in which TS_max_ is the highest averaged TS value of all the pairwise comparisons between each specimen and *G. catenatum* for the corresponding photosynthesis-related gene.

To investigate whether high or low presence of photosynthetic components could simply be explained by high or low BUSCO coverage, the transcriptomic representation of certain sections of the photosynthetic pathway was compared to the BUSCO ortholog transcript presence. This comparison was performed once for the combined mechanisms of *ptox*, cytochrome b_6_f, PSI, and PET (14 genes) and a second time for ATP synthase (eight genes). For each specimen, the comparison was calculated by finding the difference between the percentage of genes represented by transcript presence for the relevant photosynthesis component and the percentage of genes represented (combining complete and fragmented sequences) in the BUSCO analysis. If the component percentage was lower than the BUSCO, it was considered reduced, and if it was higher, it was enriched.

## Supplementary Material

pgag287_Supplementary_Data

## Data Availability

The data underlying this article are available at the SRA database on NCBI (BioProject PRJNA1496452), GenBank (accessions PZ697211–PZ697252 and PZ699697–PZ699739), and Borealis (https://doi.org/10.5683/SP4/DANYQG).
