# Supplementary material for: Evolution of complex organelles in ocelloid-bearing dinoflagellates
Source: PNAS Nexus. 2026 Aug 27;5(9):pgag287. doi: 10.1093/pnasnexus/pgag287 (PMC13560084; doi:10.1093/pnasnexus/pgag287)
Supplement: Supplementary file 1 [file pgag287_supplementary_data.docx]

**SUPPLEMENTARY INFORMATION**

**Table S1. Collection and sequencing information for warnowiid single cells.** Bar chart shows BUSCO orthologous gene presence in the transcriptome, differentiating single copy (light blue), duplicated copy (dark blue), fragmented (red), and missing (grey) sequences.

**Figure S1. Light micrographs of cells isolated in this study. A)** *Olawowia* sp. **B)** *O. reka* **C)** *O. strepia* **D)** *O. habria* **E)** *Erythropsidinium scarlatinum* **F)** *Warnowia* sp. 1 **G)** *Warnowia* sp. 2 **H)** *Warnowia* sp. 3 **I)** *Warnowia* sp. 5 **J)** *Warnowia* sp. 4 **K)** *Cyklopsia gemma* **L)** *C. vigilans*. Arrows indicate ocelloids, arrowheads indicate nematocysts, and asterisks mark food vacuoles. Scale bar in B corresponds to all panels.

**Figure S2. Maximum likelihood phylogeny of 18S rRNA gene sequences.** Cells collected in this study are colored in pink. The scale bar represents the estimated number of nucleotide substitutions per site, and the model used to generate the tree is shown. Node values represent bootstrap support (1000 ultrafast), and black dots indicate a value of 100. Broken branches are shown at half their original length. Alignment can be found at https://doi.org/10.5683/SP4/DANYQG.

**Figure S3. Maximum likelihood phylogeny of 28S rRNA gene sequences.** Cells collected in this study are colored in blue. The scale bar represents the estimated number of nucleotide substitutions per site, and the model used to generate the tree is shown. Node values represent bootstrap support (1000 ultrafast), and black dots indicate a value of 100. Broken branches are shown at half their original length. Alignment can be found at https://doi.org/10.5683/SP4/DANYQG.

**Figure S4. Contaminant transcripts recovered from CU23078.** In this cell, transcripts were found that consistently clustered with *Paragymnodinium shiwaense* or *Polykrikos lebouriae* in the Gymnodiniales, branching mostly outside of the warnowiid clades. These transcripts were determined to be derived from a recently consumed photosynthetic prey cell visible inside a food vacuole at the time of collection (**Figure S1**), as homologs from both the warnowiid and the closely related prey were found coexisting for some genes. Subclades of eight single gene phylogenies are shown that contain all warnowiid sequences from the corresponding tree. Warnowiid sequences are colored blue and contaminant sequences are red. A non-contaminant sequence from CU23078 is highlighted in orange, coexisting with a contaminant sequence from the same transcriptome. Node values represent bootstrap support (1000 ultrafast), and black dots indicate a value of 100. Alignments for all trees can be found at https://doi.org/10.5683/SP4/DANYQG.

**Figure S5. ATP synthase homologs and twin-arginine translocation (TAT) pathway transcripts.** Numbers indicate the number of transcripts recovered for each genus. Red corresponds to mitochondrial homologs and purple is vacuolar. Bar graph represents BUSCO coverage for all combined transcriptomic data for each genus. Extracted transcripts available at https://doi.org/10.5683/SP4/DANYQG.

**Figure S6. Phylogenomic representation of divergence in sparsely expressed genes.** All non-contaminant warnowiid sequences are colored green. The broken branch is depicted at half its true length. The scale bars represent the estimated number of amino acid substitutions per site for their respective trees, and the model used to generate each tree is shown. Node values represent bootstrap support (1000 ultrafast), and black dots indicate a value of 100. Alignments for trees can be found at https://doi.org/10.5683/SP4/DANYQG.

**Figure S7. Maximum likelihood phylogeny of rhodopsins.** Warnowiids are colored pink, and all other dinoflagellates are colored blue. Grey highlighted regions show the different eukaryotic functional types of rhodopsin. The scale bar represents the estimated number of amino acid substitutions per site, and the model used to generate the tree is shown. Node values represent bootstrap support (1000 ultrafast), and black dots indicate a value of 100. Alignment can be found at <https://doi.org/10.5683/SP4/DANYQG>.

**TAXONOMIC SUMMARY**

Class Dinophyceae Fritsch, 1927

Order Gymnodiniales Lemmermann, 1910

Family Warnowiaceae Lindemann, 1928

**Genus *Olawowia* gen. nov. Cooney & Keeling, 2026**

**Diagnosis.** Heterotrophic ocelloid-bearing dinoflagellates that form the sister lineage to *Erythropsidinium* and lack nematocysts. Cells are generally elongated in shape, with a transverse flagellum that encircles the cell three times. The ocelloid is positioned at or anterior to the midpoint of cells in interphase, and has a black-appearing ocelloid plastid under bright field.

**Type species.** *Olawowia reka* Cooney & Keeling 2026

**Etymology.** *Olawowia* is a combination of the Papiamento words “ola” (wave) and “wowo” (eye). Gender is feminine.

**Zoobank ID.** urn:lsid:zoobank.org:act:3D9B6836-A533-4184-BFB1-7F8BA3A57FF5

***Olawowia reka* sp. nov. Cooney & Keeling, 2026**

**Diagnosis.** Elongated heterotrophic ocelloid-bearing dinoflagellate. Vegetative cells are approximately 200 µm long and 50 µm wide, with uniform width across the cell, tapering into round anterior an posterior ends. The ocelloid is anteriorly-facing, positioned in the anterior third of the cell, and 40 µm in length. Cells sometimes have small, peripherally located pinkish pigmented inclusions.

**Sequence data.** Raw reads: SRA (project number PRJNA1496452). 18S rRNA gene sequence: PZ697238.

**Type locality**. The specimen was collected in Curacao (12.1227, -68.9695) at surface depth.

**Type habitat.** Marine.

**Holotype.** Cell CU24059 shown in Fig. S1B.

**Etymology.** The species name “reka” is derived from the Papiamento word, “rèk”, which translates to “stretch”, referencing the elongated shape of members of this species. Gender is feminine.

**Zoobank ID.** urn:lsid:zoobank.org:act:9EDCE81D-148D-4DF9-B580-2988D4FA0E9A

***Olawowia habria* sp. nov. Cooney & Keeling, 2026**

**Diagnosis.** Heterotrophic ocelloid-bearing dinoflagellate. Vegetative cells are 75-90 µm long and 35-45 µm wide, and are generally unpigmented. Cells are quenelle shaped with blunt pointed ends. The ocelloid is anteriorly-facing, positioned in the anterior half of the cell, and 20 µm in length.

**Sequence data.** Raw reads: SRA (project number PRJNA1496452). 18S rRNA gene sequence: PZ697234.

**Type locality**. The specimen was collected in Curacao (12.1248, -68.9683) at surface depth.

**Type habitat.** Marine.

**Holotype.** Cell CU23071 shown in Fig. S1D.

**Etymology.** The species name “habria” is derived from the Papiamento word, “habrí”, which translates to “open”. Gender is feminine.

**Zoobank ID.** urn:lsid:zoobank.org:act:5A0A3218-6E90-4843-B224-A6BAFED2713E

***Olawowia strepia* sp. nov. Cooney & Keeling, 2026**

**Diagnosis.** Heterotrophic ocelloid-bearing dinoflagellate. Vegetative cells are approximately 125 µm long and 75 µm wide. Cell shape is asymmetrical and bent, with a conical anterior end, and an angular posterior. The cingulum presents as a pronounced ridge that encircles the cell. Striations are sometimes visible, running longitudinally along the cell. Cells are mostly unpigmented, with the exception of small, dark, peripheral inclusions, appearing stripe-like, as they follow the striations and cingular ridge of the cell.

**Sequence data.** Raw reads: SRA (project number PRJNA1496452). 18S rRNA gene sequence: PZ697240.

**Type locality**. The specimen was collected in Curacao (12.1248, -68.9683) at surface depth.

**Type habitat.** Marine.

**Holotype.** Cell CU23086 shown in Fig. S1C.

**Etymology.** The species name “strepia” is derived from the Papiamento word, “strepiá”, which translates to “striped”, referencing the dark inclusions on these cells, resembling stripes. Gender is feminine.

**Zoobank ID.** urn:lsid:zoobank.org:act:5470B82A-46EF-4DE5-A8C0-EF3F20F3E519

Genus *Erythropsidinium* Silva, 1960

***Erythropsidinium nutkana* sp. nov. Cooney & Keeling, 2026**

**Diagnosis.** An ocelloid-bearing warnowiid dinoflagellate with a cell body approximately 55 µm by 45 µm. Cells have diffuse pink pigmentation, sometimes highly saturated in color, reaching a shade of magenta, but some cells can be only faintly pigmented. Vegetative cells bear a piston that protrudes from the posterior end of the cell, and when extended, this appendage can be at least four times the length of the cell body, and sometime much longer. The shape of the cell body is distinct from other *Erythropsidinium*, as it is slightly rounded in shape. The widest point of the cell at its midpoint. The posterior end of the cell is narrows, and the posterior face of the cell is flat. The anterior half of the cell also narrows from the midpoint, terminating at the anterior end in a pointed, but blunt apex. During interphase, the ocelloid is located in the anterior half of the cell, oriented in an anterior direction. The transverse flagellum encircles the cell, but does not terminate in a tight spiral at the anterior end of the cell, as seen in other species.

**Sequence data.** Raw reads: SRA (project number PRJNA922587). 18S rRNA gene sequence: OP604721.

**Type locality**. The specimen was collected on Quadra Island (50.1151, -125.2207) at surface depth.

**Type habitat.** Marine.

**Holotype.** Cell shown in Cooney et. al 2023, Fig. 2R.

**Etymology.** The species is named after *Rosa nutkana*, the wild rose native to the west coast of Canada that is a similar shade of pink. Gender is feminine.

**Zoobank ID.** urn:lsid:zoobank.org:act:9C152ABB-994C-4387-ABA2-E053E7FFCD6F

Genus *Cyklopsia* Cooney, Leander, & Keeling, 2021

**Type species.** Cyklopsia gemma Cooney, Leander & Keeling, 2021

***Cyklopsia vigilans* (Marshall, 1925) comb. nov.**

**Basionym.** *Proterythropsis vigilans* Marshall, 1925

**Synonym.** *Nematopsides vigilans* (Marshall, 1925) sensu Greuet, 1973

**Zoobank ID.** urn:lsid:zoobank.org:act:6BEAA519-6BFA-4F24-8F9E-3230F057249A
